# RNA sequencing of mesenchymal stem cells reveals a blocking of differentiation and immunomodulatory activities under inflammatory conditions in rheumatoid arthritis patients

**DOI:** 10.1186/s13075-019-1894-y

**Published:** 2019-05-06

**Authors:** Jose Ramon Lamas, Benjamin Fernandez-Gutierrez, Arkaitz Mucientes, Fernando Marco, Yaiza Lopiz, Juan Angel Jover, Lydia Abasolo, Luis Rodríguez-Rodríguez

**Affiliations:** 1 Instituto de Investigación Sanitaria del Hospital Clínico San Carlos (IdISSC), UGC de Reumatología, Hospital Clínico San Carlos, Madrid, Spain; 2 Instituto de Investigación Sanitaria del Hospital Clínico San Carlos (IdISSC), UGC de Traumatología, Hospital Clínico San Carlos, Madrid, Spain

**Keywords:** Mesenchymal stem cells, Rheumatoid arthritis, RNA-seq, Immunomodulation

## Abstract

**Introduction:**

Mesenchymal stem cells (MSCs) have the ability to differentiate into different types of cells of the mesenchymal lineage, such as osteocytes, chondrocytes, and adipocytes. It is also known that under inflammatory stimuli or in the appropriate experimental conditions, they can also act as regulators of inflammation. Thus, in addition to their regenerating potential, their interest has been extended to their possible use in cell therapy strategies for treatment of immune disorders.

**Objective:**

To analyze, by RNA-seq analysis, the transcriptome profiling of allogenic MSCs under RA lymphocyte activation.

**Methods:**

We identified the differentially expressed genes in bone marrow mesenchymal stem cells after exposure to an inflammatory environment. The transcriptome profiling was evaluated by means of the precise measurement of transcripts provided by the RNA-Seq technology.

**Results:**

Our results evidenced the existence of blocking of both regenerative (differentiation) and immunomodulatory phenotypes under inflammatory conditions characterized by an upregulation of genes involved in immune processes and a simultaneous downregulation of genes mainly involved in regenerative or cell differentiation functions.

**Conclusions:**

We conclude that the two main functions of MSCs (immunomodulation and differentiation) are blocked, at least while the inflammation is being resolved. Inflammation, at least partially mediated by gamma-interferon, drives MSCs to a cellular distress adopting a defensive state. This knowledge could be of particular interest in cases where the damage to be repaired has an important immune-mediated component.

**Electronic supplementary material:**

The online version of this article (10.1186/s13075-019-1894-y) contains supplementary material, which is available to authorized users.

## Introduction

Rheumatoid arthritis (RA) is a chronic, systemic inflammatory disease with a wide spectrum of clinical manifestations, varying from mild to very severe [1, 2]. In order to prevent serious long-term complications, such as joint destruction, functional loss, and preterm mortality, disease remission is the current treatment goal for this condition [3]. However, most *patients* do not achieve this state [4, 5], despite the use of new drugs, such as biologic agents. Therefore, new therapies are needed to reduce the burden of this condition.

Bone marrow (BM)-derived mesenchymal stem cells (MSCs) are plastic adherent and self-replicating adult stem/progenitor cells with multipotential capacities. MSCs were initially identified in bone marrow but are present virtually in any tissue. The most common sources, besides bone marrow, are adipose tissue, umbilical cord and cord blood, synovial tissue, and dental pulp [6].

MSCs can be easily isolated and are capable to undergo osteogenic, chondrogenic, and adipogenic differentiation in vitro. More recently, it has been also described in vitro the potential of MSCs to interact with immune cells and display immunomodulatory and anti-inflammatory properties. Thus, the use of MSCs as therapeutic agents has been expanded offering new perspectives beyond their regenerative potential becoming optimal candidates for the treatment of immune-mediated diseases [6].

Interactions between MSCs and immune cells are characterized by the existence of a bi-directional crosstalk which influences the final outcome. It is mediated by a combination of cell-to-cell interactions and by paracrine secretion of different soluble factors [7, 8]. These include the interleukin-10 (IL-10) [9], the inducible nitric oxide synthase (iNOS), the cyclooxygenase-2 (COX-2 [10, 11]), the transforming growth factor-β (TGF-β) [12], the prostaglandin E2 (PGE2), the nitric oxide (NO), and the indoleamine 2,3,dioxygenase (IDO), among others [13].

Altogether, these combined effects determine the triggering of several immune functions after stimulation by proinflammatory mediators, including the polarization of macrophage pro-inflammatory phenotype M1 to the immunoregulatory M2 phenotype [14]. M2 polarization, in turn, increases the Th2 response (Treg upregulation, immunosuppression, and tissue remodeling). Additionally, MSCs also can enhance angiogenesis promoting the VEGF and angiopoietin-1 production [15].

We used RNA sequencing (RNA-Seq) as an experimental approach to perform a precise measurement of transcripts generated by BM-MSCs interacting with activated or inactivated peripheral blood mononuclear cells (PBMCs); moreover, we classified the highly regulated genes from both groups according to functional gene ontologies (GOs) in order to gain insight into the changes these cells undergo when exposed to an inflammatory environment. This knowledge is fundamental for a better understanding of the biological interactions between MSCs and the immune system and to progress toward clinical application of MSCs in regenerative medicine and cell therapy strategies.

## Materials and methods

### Patients and donors

Demographic and clinical characteristics BM-MSCs donors and of the RA patients are shown in Additional file 1: Table S1 and Additional file 2: Table S2, respectively.

### Bone marrow mesenchymal stem cells

Mesenchymal stem cells (MSCs) were obtained from bone marrow (BM) aspirates collected from the iliac crests of three donors, following informed consent. We included subjects older than 18 years old, with no previous diagnosis of autoimmune disease or lymphoproliferative/neoplastic conditions. Briefly, aspirates were diluted in an equal volume with saline and centrifuged over a Ficoll layer at 2000×*g* for 20 min. Cellular fraction recovered was washed two times in Dulbecco’s modified Eagles medium (DMEM) (Lonza). The cell pellet obtained was suspended in 5 ml with complete culture medium (DMEM supplemented with 2 mM glutamine, 0.06% penicillin, 0.02% streptomycin, and 10% FBS). Cultures were incubated at 37 °C in a 5% CO_2_ humidified atmosphere in 25-cm^2^ flasks. After several days, non-adherent cells were removed and fresh medium was added. The medium was exchanged every 4 days of culture. When cultured cells reached 80–90% confluence, adherent cells were trypsinized (0.05% trypsin/1.0 mM EDTA), harvested, and expanded in 25-cm^2^ flasks. MSC characterization was performed according to the minimal criteria recommended by the ISCT (International Society for Cellular Therapy) described by Dominici et al [16]. Cells in the fourth passage were used in the experimental analysis.

Bone marrow aspirates and blood were obtained in accordance with Good Clinical Practices and the principles expressed in the Declaration of Helsinki. The study was approved by our institutional Ethics Committee (Comité Ético de Investigación Clínica Hospital Clínico San Carlos—Madrid).

### Isolation of PBMCs from RA patients

Five consecutive patients diagnosed with RA according to the 2010 ACR/EULAR criteria, attending the Hospital Rheumatology Outpatient Clinic, were included in this study. Patients were over the age of 18 at disease onset and had no previous history of any other chronic disease such as diabetes, chronic kidney disease, and/or lymphoproliferative/neoplastic conditions. We excluded from the study those patients receiving more than 10 mg/day of prednisone or equivalent, those who had received any intramuscular dose of corticosteroids in the previous 2 months, or those treated with drugs that can affect lymphocyte activation, such as calcium antagonists or statins. At inclusion, demographic and clinical data were collected, and a fasting venous blood sample was extracted on EDTA as anticoagulant. PBMCs were separated by centrifugation on a Ficoll-Hypaque gradient at 900×*g*, for 20 min at 25 °C.

### In vitro BM-MSC–PBMC co-cultures

BM-MSCs (2 × 10^5^ cells) were cultured alone for 24 h in non-treated Falcon® 6-wells flat bottom plates (Corning) in complete low-glucose DMEM (Lonza Group Ltd., Basel, Switzerland) at 37 °C and 5% CO_2_. After this time, the medium was replaced by supplemented RPMI. PBMCs (2 × 10^6^ cells) were added to the wells in ratio 1:10 (BM-MSC:PBMCs) mostly based on cellular and cellular membrane size.

In some wells, anti-CD3-/anti-CD28-coated beads (Dynabeads® Human T-Activator; Life Technologies) were also added at a ratio of 1 bead per 4 PBMCs.

Three days after co-culturing, supernatant was collected removing the anti-CD3-/anti-CD28-coated beads as well as PBMCs not attached to the BM-MSCs. Furthermore, those weakly attached to the surface of the BM-MSCs were collected by gently pipetting the bottom of each plate with clean RPMI. CD3/CD28 beads were magnetically removed (following the manufacturer’s protocol), and PBMCs were recovered after centrifugation and counted, and their viability was assessed by Trypan blue staining. BM-MSCs were treated with Trypsin/EDTA and the remaining PBMCs attached were removed by using anti-CD45 antibodies conjugated to paramagnetic microbeads (Miltenyi Biotec, Spain). Purified BM-MSCs were stored in RNA protect solution (Qiagen Iberia, Madrid, Spain) at − 80 °C.

### RNA extraction and processing

Total RNA from BM-MSCs was extracted using the RNeasy Mini Kit (Qiagen Iberia, Madrid, Spain), according to the manufacturer’s protocol. RNA concentration was quantified spectrophotometrically (Nanodrop ND-1000, Wilmington, DE), and its quality and integrity assessed by capillary electrophoresis on an Agilent 2100 Bioanalyzer (Agilent Technologies. Spain). Barcoded cDNA libraries were prepared from poly(A) enriched mRNA using NebNext Ultra Directional RNA Library Prep Kit (New England Biolabs, Spain). Pooled libraries were sequenced on an Illumina NextSeq 500 instrument to generate on average 32.9 million single-end reads of 76 bp length. To avoid a batch effect bias, all samples were run simultaneously twice, and results merged.

#### Assessment of PBMC RNA contamination

In order to assess the degree of RNA contamination from the PBMCs, different approaches were followed (see Additional file 4).

### Bioinformatic analyses

Raw sequence quality control was performed using FastQC [17] (Babraham Bioinformatics). The raw sequence reads (FASTQ format) were aligned to the cDNA sequences of the human GRCh37 reference assembly available in UCSC Genome Browser (http://hgdownload.soe.ucsc.edu/goldenPath/hg19/bigZips/hg19.2bit), using the Rsubread Bioconductor package v1.20.3 [18], using the default settings, and reporting only uniquely mapped reads.

Read summarization was performed with featureCounts [19], using the in-built gene annotations from the NCBI RefSeq for Hg19, included in the Rsubread package. Mapped reads for each sample were summarized into the meta-feature “gene,” thus obtaining gene-level expression counts that were used as input for gene expression analysis.

In order to improve the statistical power by decreasing the number of multiple comparisons to adjust for and to reduce the possible bias of very small counts with no biological significance, we removed those genes with low expression according to the number of counts for million mapped reads (CPMs). We set a threshold of at least 1 count per million (CPM), in at least 4 of the 5 active and/or 4 of the 5 resting samples.

To adjust for variable sequencing depths between samples, the raw gene counts were normalized using a weighted trimmed mean of the log expression ratios (Trimmed Mean of *M* values [TMM] algorithm) as implemented in the edgeR Bioconductor package [20, 21]. A multidimensional scaling (MDS) plot was used as an unsupervised approach to visualize the data structure of the analyzed samples.

### Statistical analysis

**Fig. 1:**
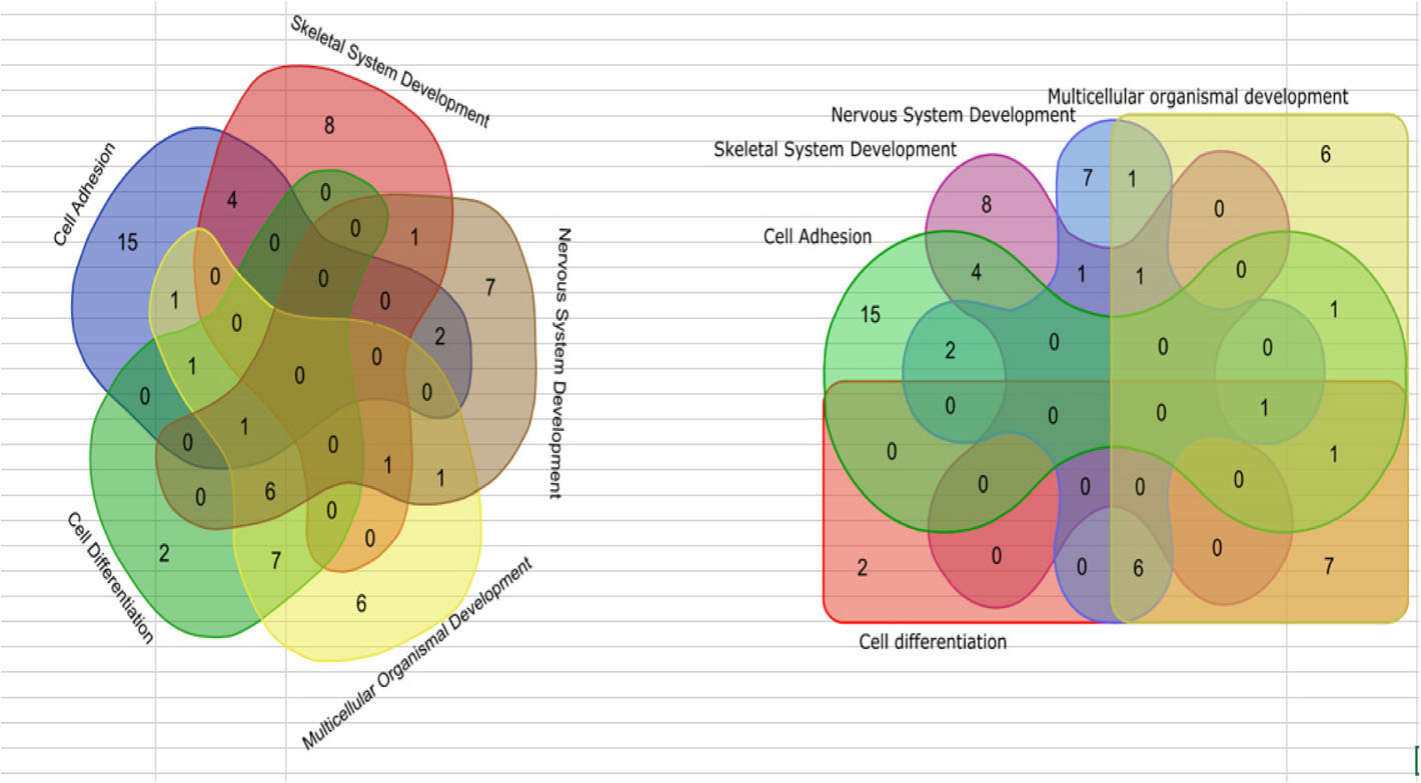
A representative diagram regarding the analysis of downregulated genes. GO downregulated terms are showed

The edgeR Bioconductor package [20] was used to identify genes that were differentially expressed between BM-MSCs co-cultured with activated or resting PBMCs. Our study design had three experimental factors: the BM-MSC donor (three healthy donors), the PBMCs donors (five RA patients), and the PBMC activation state (two levels: resting or activated). Therefore, this comparison (exposure to resting or activated PBMCs from RA patients) was nested by the RA patient from whom the PBMCs were obtained and, in turn, nested by the BM-MSCs donor (Fig. 1).

Based on this design, we used the following formula:

$$ \sim \mathrm{MSC}\_\mathrm{ID}+\mathrm{MSC}\_\mathrm{ID}:\mathrm{RA}\_\mathrm{ID}+\mathrm{MSC}\_\mathrm{ID}:\mathrm{Activ} $$

Due to the unbalanced design, we manually edited the matrix generated by this formula, removing those columns containing no information. Differential expression was analyzed using the quasi-likelihood *F* test [20]. Significant differentially expressed genes (DEGs) were defined as those with a log_2_ fold change ≥ 2 and a false discovery rate (FDR) of ≤ 1% (adjusted with the Benjamini-Hochberg method [22]).

### Quality of RNA extraction

The mean (SD) RNA concentration was 255.9 (147.4) ng/μl for BM-MSCs co-cultured with resting PBMCs and 78.7 (28.5) ng/μl for BM-MSCs co-cultured with activated PBMCs. We used 250 ng of RNA from each sample. All samples had a RNA integrity number of 10.

After sequencing, we generated a total number of unique reads mapped to the human genome between 29.6 and 34.9 million, with a mean (SD) across samples of 32.9 (1.7) millions. These reads were summarized into gene-level expression counts, resulting in a mean (SD) of 23.8 (1.5) million successfully assigned reads [a mean (SD) percentage of 72.2 (1.7)] (Table 3). The difference in the number of reads between BM-MSCs co-cultured with activated or resting PBMCs was not statistically significant (Student’s *t* test, *p* = 0.82), nor was the difference in the number of reads among BM-MSC samples regarding the donor (ANOVA *p*-value = 0.66).

Initial summarization of reads into genes revealed 25,702 metafeatures. Count data was filtered based on the number of CPMs in that particular sample. We set a CPM threshold of 1, which represented a minimum gene count between 22 and 26, depending on the library size. After filtering, 12,821 genes remained. We further removed those genes lacking Gene Symbol identifier (*n* = 219). Therefore, 12,602 genes were analyzed for differential expression.

The MDS plot clearly distinguishes the transcriptional profiles of those BM-MSCs exposed to resting or activated PBMCs, separated along the first dimension. Furthermore, along the second dimension, a difference between the BM-MSCs from the first donor and those from donors 2 and 3 was observed, regardless the BM-MSCs had been exposed to active or resting PBMCs.

### Gene Ontology analysis

To understand the biological impact of the gene expression changes, we performed functional enrichment analysis. Considering that for RNA sequencing data, gene length and read count can introduce biases in the gene ontology (GO) enrichment analysis, we used GOseq [23] in order to minimize this bias. We manually introduced the gene length, based on the data from the inbuilt NCBI RefSeq Hg19 annotation from the Rsubread package. We analyzed separately those genes up- or downregulated, considering significantly enriched those terms with an FDR ≤ 1%. Furthermore, since we expected to observe overlapping themes, we collapsed these terms into “supra-categories.”

### Network analysis

To further analyze the biological impact of the DEGs, we created protein-protein interaction networks using the STRING database [24]. In order to assess only those interactions more likely to take place, we used a confidence score cutoff of 900, required experimental evidence in order to consider protein-protein interaction, and used only first-order interactions (meaning only molecules directly interacting with our DEG genes).

We also used the InnateDB [25], another curated database of experimentally verified proteins interactions and signaling pathways involved in the innate immune response.

#### GBP5 analysis

##### Co-culture experiments

Bone marrow-derived MSCs (BM-MSC) from 2 donors and peripheral blood lymphocytes (PBL) from 4 donors were co-cultured on transwell 12-well culture plates (#3460, Corning® Transwell®) in RMPI 1640 medium (#BE12-167F, Lonza®) supplemented with 10% FBS and antibiotics at 37 °C in a 5% CO2 atmosphere. In each well, 100,000 MSCs were seeded and then added 800,000 PBLs in the upper chamber. Previously, when needed, PBLs were activated with Dynabeads® Human T-Activator CD3/CD28 for T Cell Expansion and Activation (#11131D, Gibco®). Finally, INF-ϒ was neutralized using anti-INF-ϒ antibody (10 μl/ml, B27, BioLegends®)

Five conditions were analyzed: BM-MSCs with anti-IFN-ϒ, BM-MSCs with PBLs, BM-MSCs with PBLs and anti-IFN-ϒ antibody, BM-MSCs with activated PBLs, and BM-MSCs with activated PBLs and anti-IFN-ϒ antibody.

##### Quantitative PCR

Following 3 days of co-culture, medium and PBL were removed and RNA from MSCs were extracted using a commercial RNA extraction kit (SPEEDTOOLS Total RNA Extraction Kit, #21.212-4210, Biotools®). For cDNA generation, Superscript® VILO® cDNA Synthesis Kit (#11754050, Invitrogen®) was employed. Finally, cDNA was resuspended in 20 μl nuclease-free water and stored at − 20 °C until required for PCR.

The expression of three genes was quantificated with specific TaqMan® assays: 18S (Hs99999901_s1, #4331182), ACTB (Hs99999903_m1, #4453320), and GBP5 (Hs00369472_m1, #4448892). Quantitative PCR was carried out following master mix indications (TaqMan® Fast Advanced Master Mix, #4444557, Applied Biosystems®) on a MasterCycler RealPlex^4^ PCR System (Eppendorf). A triplicate of each sample was done. Fold change of GBP5 for each condition was calculated via 2^−ΔΔCt^ method.

## Results

### Differential expression of genes

Based on the thresholds set for fold-change and *p* value, comparing the transcriptomes of BM-MSCs samples exposed to activated RA PBMCs and exposed to resting RA PBMCs, we observed 847 DEGs in total, with 236 genes downregulated and 611 genes upregulated (Table 1 and Additional file 3: Genes). Of those DEGs, 321 were not expressed in BM-MSCs co-cultured with resting PBMCs, and their expression was induced by exposure to active PBMCs. Conversely, the expression of 87 genes was abrogated after exposure to activated PBMCs. Four hundred thirty-nine genes showed expression in both groups.

**Table 1:** Top 20 most significantly differentially expressed genes when comparing the transcriptomes of the bone marrow mesenchymal stem cells (BM-MSCs) exposed to activated peripheral blood mononuclear cells (PBMCs) from rheumatoid arthritis patients and BM-MSCs exposed to resting PBMCs from RA patients. Positive logarithm of the fold change (Log2FC) indicates greater expression on BM-MSCs exposed to activated PBMCs

Entrez ID	Symbol	Log2FC	FDR
4317	MMP8	11.4	3.74E−05
3620	IDO1	9.8	3.74E−05
115362	GBP5	9.5	3.74E−05
3055	HCK	8.4	3.74E−05
5452	POU2F2	6.5	3.74E−05
1440	CSF3	12.4	4.66E−05
6364	CCL20	9.9	7.44E−05
3553	IL1B	9.7	7.44E−05
84419	C15orf48	9.2	7.44E−05
64127	NOD2	9.0	7.44E−05
115361	GBP4	6.5	7.44E−05
272	AMPD3	5.7	7.44E−05
9235	IL32	5.2	7.44E−05
6352	CCL5	8.5	9.68E−05
261729	STEAP2	5.2	1.06E−04
2537	IFI6	4.6	1.06E−04
4939	OAS2	3.7	1.08E−04
2919	CXCL1	10.3	1.15E−04
165904	XIRP1	8.1	1.15E−04
29015	SLC43A3	4.8	1.15E−04

### Biological interpretation of the differentially expressed genes

Regarding the analysis of up-regulated genes, 960 lacked GO annotation and were excluded from the analysis (47 out of 611 DEGs and 913 out of 11,991 non-DEGs). We observed 764 biological process (BP) GO terms significantly overrepresented, considering a FDR threshold < 1%. The 20 most significant GO terms are shown in Table 2 and Additional file 3: Genes.

**Table 2:** Top 20 most significantly overrepresented biological process gene ontology terms in upregulated differentially expressed genes when comparing the bone marrow mesenchymal stem cells (BM-MSCs) exposed to activated peripheral blood mononuclear cells (PBMCs) from rheumatoid arthritis patients vs. BM-MSCs exposed to resting PBMCs from RA patients

Category	Term	Genes in category	DEGs in category	*p* value	FDR *p* value
GO:0006955	Immune response	1014	187	6.66E−65	1.25E−60
GO:0006952	Defense response	1109	192	4.23E−62	3.98E−58
GO:0002376	Immune system process	1669	227	2.41E−55	1.51E−51
GO:0043207	Response to external biotic stimulus	571	121	1.46E−46	5.49E−43
GO:0051707	Response to other organism	571	121	1.46E−46	5.49E−43
GO:0034097	Response to cytokine	613	125	2.84E−46	8.92E−43
GO:0071345	Cellular response to cytokine stimulus	531	115	3.89E−45	1.05E−41
GO:0009607	Response to biotic stimulus	595	121	1.35E−44	3.19E−41
GO:0006954	Inflammatory response	366	95	2.48E−44	5.19E−41
GO:0019221	Cytokine-mediated signaling pathway	422	101	1.70E−43	3.19E−40
GO:0002684	Positive regulation of immune system process	618	117	5.97E−40	1.02E−36
GO:0050896	Response to stimulus	5154	398	7.48E−40	1.17E−36
GO:0002682	Regulation of immune system process	979	146	7.82E−38	1.13E−34
GO:0009605	Response to external stimulus	1588	188	1.10E−35	1.38E−32
GO:0045087	Innate immune response	708	119	1.70E−35	1.99E−32
GO:0034341	Response to interferon-gamma	103	46	3.07E−33	3.04E−30
GO:0009617	Response to bacterium	298	74	5.57E−33	5.25E−30
GO:0006950	Response to stress	2812	260	3.03E−32	2.72E−29
GO:0007154	Cell communication	3731	304	1.14E−29	9.35E−27
GO:0001775	Cell activation	600	100	1.66E−29	1.24E−26

Regarding the analysis of downregulated genes, 960 lacked GO annotation and were excluded from the analysis (9 out of 236 DEGs and 951 out of 12,366 non-DEGs). We observed 26 BP GO terms significantly overrepresented, considering a FDR threshold < 1%. The 20 most significant GO terms are shown in Fig. 1, Table 3, and Additional file 3: Genes.

**Table 3:** Top 20 most significantly overrepresented biological process gene ontology terms in downregulated differentially expressed genes when comparing mesenchymal stem cells (MSCs) exposed to activated peripheral blood mononuclear cells (PBMCs) from rheumatoid arthritis (RA) patients with MSCs exposed to resting PBMCs from RA patients

Category	Term	Genes in category	DEGs in category	*p* value	FDR *p* value
GO:0030198	Extracellular matrix organization	280	27	2.51E−11	7.88E−08
GO:0043062	Extracellular structure organization	280	27	2.51E−11	7.88E−08
GO:0032501	Multicellular organismal process	4070	128	1.65E−10	4.44E−07
GO:0007275	Multicellular organism development	3185	108	3.75E−10	7.84E−07
GO:0001501	Skeletal system development	357	28	8.09E−10	1.52E−06
GO:0044707	Single-multicellular organism process	3819	120	1.51E−09	2.36E−06
GO:0048731	System development	2834	98	1.77E−09	2.37E−06
GO:0009888	Tissue development	1147	54	2.54E−09	3.19E−06
GO:0048856	Anatomical structure development	3356	109	3.40E−09	4.00E−06
GO:0003008	System process	810	43	4.52E−09	5.00E−06
GO:0032502	Developmental process	3770	117	7.01E−09	7.33E−06
GO:0044767	Single-organism developmental process	3721	115	1.47E−08	1.46E−05
GO:0007155	Cell adhesion	890	44	2.26E−08	2.09E−05
GO:0022610	Biological adhesion	893	44	2.47E−08	2.09E−05
GO:0022617	Extracellular matrix disassembly	94	13	8.97E−08	6.49E−05
GO:0048513	Animal organ development	1980	72	1.20E−07	8.39E−05
GO:0030574	Collagen catabolic process	57	10	2.43E−07	1.63E−04
GO:0044243	Multicellular organism catabolic process	60	10	4.05E−07	2.54E−04
GO:0009653	Anatomical structure morphogenesis	1891	68	6.10E−07	3.59E−04
GO:0009887	Organ morphogenesis	617	32	1.76E−06	9.74E−04

Based on the ancestry relations between GO terms, we grouped them in 9 supra-categories (Table 4). The supra-category “Immune system-related” included BP GO terms associated with immune cell activation, differentiation, proliferation, aggregation, apoptosis, and cytotoxicity. Furthermore, we also included those terms related with chemotaxis and chemokine/cytokine production, secretion, and response.

**Table 4:** Supra-categories including the biological process gene ontology terms significantly overrepresented when comparing mesenchymal stem cells (MSCs) exposed to activated peripheral blood mononuclear cells (PBMCs) from rheumatoid arthritis (RA) patients vs. MSCs exposed to activated PBMCs from RA patients

Supra-category	Number of categories in upregulated genes	Number of categories in downregulated genes
Immune system-related	407	2
Signaling-related	89	0
Calcium metabolism-related	53	0
Development-related	50	18
Response to pathogens-related	45	0
Nucleic acids metabolism-related	18	0
Wound repair-related	12	0
Nitric oxide metabolism-related	6	0
Miscellanea	84	6

We also performed network analysis, in order to identify potentially affected biological functions and molecular networks in BM-MSCs in response to activated PBMCs. Our DEGs were mapped to two molecular interaction databases: STRING and InnateDB. Regarding the first one, 27 subnetworks with at least 3 nodes were observed. We observed 94 different modules. After Bonferroni correction (threshold *p* value = 5 × 10^−4^), 31 remained significant, comprising between 10 and 98 genes each. When enrichment analyses were performed in each of the significant modules, we observed 569 BP GO terms significantly upregulated, considering a FDR *p* value < 0.05.

Regarding the second database, first-order interactions returned subnetworks with too many nodes (Subnetwork 1: 5482 nodes, 13,927 edges and 953 seeds). Therefore, in order to reduce the complexity of the network, we used zero-order interactions. We observed 37 different modules. After Bonferroni correction (threshold *p* value = 1.4 × 10^−3^), 3 remained significant, comprising between 14 and 30 genes each. When enrichment analyses were performed in each of the significant modules, we observed 152 BP GO terms significantly upregulated, considering a FDR *p* value < 0.05**.**

### GBP5 analysis

**Fig. 2:**
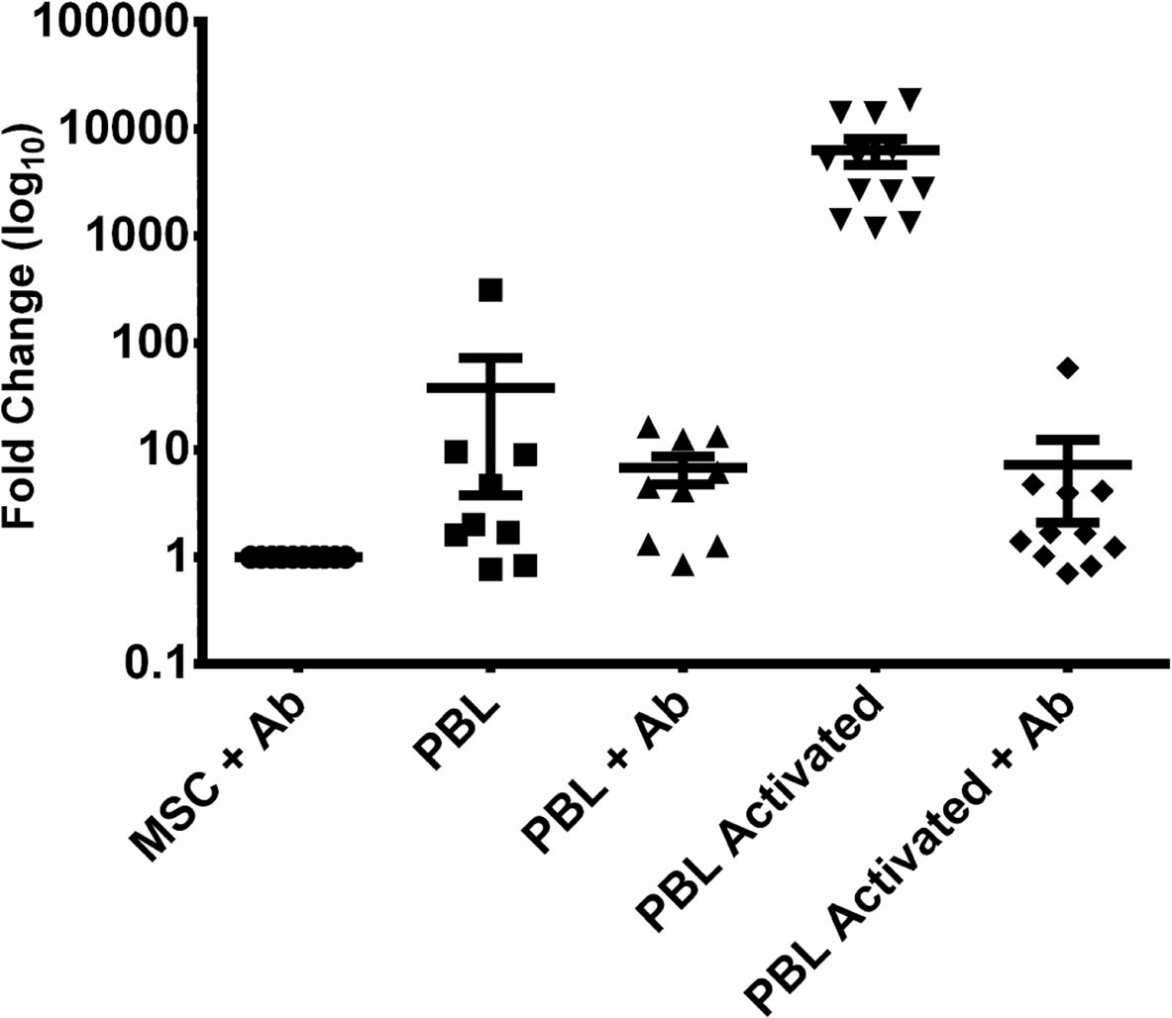
Quantitative PCR related to GBP5 on MSCs in different transwell co-culture conditions. Fold change is showed. MSC mesenchymal stem cells, PBL peripheral blood lymphocytes, Ab anti-IFNgamma

To gain insight into the mechanisms related to these results, we performed transwell co-cultures between PBMCs and MSCs. As we can clearly observe in Fig. 2, GBP5 expression is overrepresented on MSCs in the presence of activated PBMCs and this expression is mediated, at least in part, by IFN-gamma.

## Discussion

MSCs are considered optimal candidates for their therapeutic application in many of the pathologies affecting the musculoskeletal system, including tendinopathies, bone fractures, or osteoarthritis. The mode of application is usually local (non-systemic) aiming to improve the regeneration of target tissue. How this is achieved depends at least on three alternative mechanisms: (1) through differentiation of the MSCs to the damaged cell type in the tissue; (2) activating endogenous progenitor cells to promote angiogenesis, by means of paracrine secretion of factors; and (3) by controlling and modulating the inflammatory response in order to facilitate reparative processes [26].

In the case of RA, these cells hold a great potential for disease amelioration, either due to their ability to differentiate or induce differentiation of local cells to preserve articular homeostasis, or (and probably much more efficiently) due to their capacity to induce immunomodulation in the context of diseases with immunological disturbances [27, 28].

Intravenous treatment with MSCs has demonstrated efficacy in RA both in vivo and in vitro [29, 30]. In this sense, when allogenic MSCs are intravenously infused in RA patients, they interact with RA PBMCs in order to modulate their immune function. On the other hand, the RA PBMCs also interact with those allogenic MSCs inducing cellular changes. These interactions are, at least in part, controlled by the activation state of RA PBMCs [31].

In our study, a clearly different behavior is showed on MSCs depending on the activation state of RA PBMCs. Activated RA PBMCs induce on MSCs a defensive/aggressive status characterized by inflammatory mechanisms. The five genes most upregulated in MSCs by activated PBMCs were MMP8, IDO1, GBP5, HCK, and POU2F2. The proteins encoded by these genes are related to inflammation exerting different roles, such as transcriptional activity on immunoglobulin gene promoters (POU2F2/OCT2) [32], tirosin kinase activity (HCK) [33], interferon-gamma-induced cellular factor (GBP5) [34], modulating T cell behavior (IDO1) [35] or inducing the breakdown of extracellular matrix and tissue remodeling (MMP8 [36]).

The specific significantly overrepresented GO terms were immune response, defense response, immune system process and response to external biotic stimulus. In our view, all these data are the result of cellular distress circumventing other functional mechanisms related to the regenerative process including differentiation and immunomodulation.

Similar results were obtained for genes with the highest number of connections. LYN gene (proto-oncogene, Src family tyrosine kinase) encodes a tyrosine protein kinase (involved in cellular activation) [37]. RPS4Y1 gene encodes the ribosomal protein S4 Y-linked 1, related to cellular energy. GNA15 gene encodes the G protein subunit alpha 15 also related to cellular energy [38]. PSMB10 gene encodes a member of the proteasome B-type family that is induced by interferon-gamma, as it occurs with GBP5 [38]. So, a cellular distress mediated, at least in part by gamma-interferon, would be at the origin of these results. The significant modules from the first subnetwork from the STRING interactive database are also in line with this explanation.

On the other hand, most downregulated genes on MSCs related to contact with activated RA cells were in the field of development and cell differentiation. So, these data again are in the context of cellular distress circumventing other functional mechanisms related to the regenerative process including differentiation and immunomodulation.

## Conclusions

The two main functions of MSCs (immunomodulation and differentiation) are in standby during the resolution phase of inflammation. At least partially, gamma interferon-mediated inflammation induces in MSCs a cellular distress leading to the adoption of a defensive/aggressive state. Our original approach has permitted to identify both a clue to focus on future treatments and a cytokine functionally implied in our results.

These results probably contraindicate the use of MSC treatment in the context of highly inflammatory environments and indicate the need of other different or additional treatments in these scenarios.

## Additional files

Additional file 1: **Table S1.** Demographic and clinical characteristics of the bone marrow mesenchymal stem cell donors included in this study. (DOCX 12 kb)

Additional file 2: **Table S2.** Demographic and clinical characteristics of the rheumatoid arthritis patients included in this study. (DOCX 13 kb)

Additional file 3: **Table S3.** Number of reads successfully mapped and assigned to the metafeature “gene” per sample. (XLSX 3343 kb)

Additional file 4: Supplementary Contamination Assessment. (DOCX 14 kb)

## Abbreviations

BM: Bone marrow
COX-2: Cyclooxygenase-2
DEGs: Differentially expressed genes
GNA15: G protein subunit alpha 15
GOs: Gene ontologies
IDO: Indoleamine 2,3,dioxygenase
IL-10: Interleukin-10
iNOS: Inducible nitric oxide synthase
MSCs: Mesenchymal stem cells
NO: Nitric oxide
PBMCs: Peripheral blood mononuclear cells
PGE2: Prostaglandin E2
RA: Rheumatoid arthritis
RNA-seq: RNA-sequencing
RPS4Y: Ribosomal protein S4 Y-linked 1
TGF-β: Transforming growth factor-β
