# Supplementary material for: RNA sequencing of mesenchymal stem cells reveals a blocking of differentiation and immunomodulatory activities under inflammatory conditions in rheumatoid arthritis patients
Source: Arthritis Res Ther. 2019 May 6;21:112. doi: 10.1186/s13075-019-1894-y (PMC6501285; doi:10.1186/s13075-019-1894-y)
Supplement: Supplementary file 1 — Table S1. Demographic and clinical characteristics of the bone marrow mesenchymal stem cell donors included in this study. (DOCX 12 kb) [file 13075_2019_1894_MOESM1_ESM.docx]

**Table S1**: Demographic and clinical characteristics of the bone-marrow mesenchymal stem cell donors included in this study.

| MSC ID | Gender | Age | Ancestry | Comorbidities | Treatment |
| --- | --- | --- | --- | --- | --- |
| MSC-01 | Female | 64 | Spaniard | Hypertension  Hypercholesterolemia  Asthma | ACE inhibitors  Statin  Inhaled steroids |
| MSC-02 | Male | 61 | Spaniard | Chronic obstructive  pulmonary disease | Inhaled anticholinergics |
| MSC-03 | Male | 65 | Spaniard | Hypertension  Hypercholesterolemia  Ischemic heart disease | Beta blocker  Statin  Acetylsalicylic acid |
