# Supplementary material for: RNA sequencing of mesenchymal stem cells reveals a blocking of differentiation and immunomodulatory activities under inflammatory conditions in rheumatoid arthritis patients
Source: Arthritis Res Ther. 2019 May 6;21:112. doi: 10.1186/s13075-019-1894-y (PMC6501285; doi:10.1186/s13075-019-1894-y)
Supplement: Supplementary file 2 — Table S2. Demographic and clinical characteristics of the rheumatoid arthritis patients included in this study. (DOCX 13 kb) [file 13075_2019_1894_MOESM2_ESM.docx]

**Table S2**: Demographic and clinical characteristics of the rheumatoid arthritis patients included in this study.

| RA ID | Gender | Age | RF | ACPA | Ancestry | CRP DAS28 | Prednisone  (mg/24h) | DMARD |
| --- | --- | --- | --- | --- | --- | --- | --- | --- |
| RA01 | Female | 59 | + | - | Spaniard | 2.69 | 0 | Leflunomide |
| RA02 | Female | 41 | + | + |  | 3.45 | 5 | Tocilizumab |
| RA03 | Female | 72 | - | - | Spaniard | 2.77 | 0 | Methotrexate |
| RA04 | Female | 68 | + | + | Spaniard | 2.79 | 0 | Gold salts  Sulfasalazine  Antimalarial |
| RA05 | Female | 38 | + | + | Spaniard | 4.22 | 5 | Leflunomide  Sulfasalazine  Antimalarial |

RF: Rheumatoid factor. ACPA: Anti-citrullinated peptides antibodies; CRP: C-reactive protein; DMARD: Disease modifying anti-rheumatic drug.
