# Supplementary material for: RNA sequencing of mesenchymal stem cells reveals a blocking of differentiation and immunomodulatory activities under inflammatory conditions in rheumatoid arthritis patients
Source: Arthritis Res Ther. 2019 May 6;21:112. doi: 10.1186/s13075-019-1894-y (PMC6501285; doi:10.1186/s13075-019-1894-y)
Supplement: Supplementary file 4 — Supplementary Contamination Assessment. (DOCX 14 kb) [file 13075_2019_1894_MOESM4_ESM.docx]

Supplementary Contamination Assessment

In order to assess the magnitude of the contamination from peripheral blood mononuclear cell (PBMC) RNA, several strategies were developed:

Percentage of PBMCs strongly attached to the bone marrow mesenchymal stem cells (BM-MSCs):

First, we collected and counted the PBMCs from the supernantant and those weakly attached to the surface of the BM-MSCs after detaching by gently pipetting. Next, the remaining PBMCs and BM-MSCs were detached from the plate using tripsine and counted, before magnetic separation with anti-CD45 paramagnetic beads. Numbers of cells are shown in Supplementary File Table 8. We observed an increase in the proportion of PBMCs attached to the BM-MSCs when they were activated. Based on this data we can hypothesize than the magnitude of the RNA contamination will be greater in those samples.

RT-PCR analysis of the CD45 and CD3 gene expression.

It was performed in our samples, and in samples of BM-MSCs cultured alone in RPMI during 72h, PBMCs from RA patients cultured alone in RPMI for 72h either resting or activated with anti-CD3/anti-CD28 beads (Supplementary File Table 9 and Supplementary File Figure 2).

PBMCs cultured alone showed the greater CD45 expression, followed by BM-MSCs co-cultured with activated PBMCs, BM-MSCs co-cultured with resting PBMCs and BM-MSCs cultured alone. Based on this data, CD45 expression fold change (FC) in BM-MSCs co-cultured with resting PBMCs compared with activated PBMCs cultured alone was 3.35x10-4 (i.e. the expression in the latter group was 2,941 times higher compared with the former). FC of BM-MSCs co-cultured with activated PBMCs compared with PBMCs cultured alone was 3.35x10-4 (i.e. the expression in the latter group was 210 times higher compared with the former).

Regarding CD3, its FC expression in BM-MSCs co-cultured with resting PBMCs compared with activated PBMCs cultured alone was 5.74x10-4 (i.e. the expression in the latter group was 1,742 times higher compared with the former). FC of BM-MSCs co-cultured with activated PBMCs compared with PBMCs cultured alone was 8.45x10-3 (i.e. the expression in the latter group was 118 times higher compared with the former).

RT-PCR analysis of the CD3 gene expression using a calibration curve for each condition (co-culture with resting or activated PBMCs).

We used a combination in different proportions of RNA from MSCs cultured alone and from resting or activated PBMCs cultured alone (The BM-MSC:PBMC proportion were 1:0, 0.95:0.05, 0.90:0.10, 0.85:0.15). In turn, the RNA from MSCs and from PBMCs was a combination (of the same amount) of RNA from the 3 BM-MSCs donors and the 5 RA patients, respectively. Supplementary File Table 10 shows the ΔCT of CD3 versus ATCB for both calibration curves. Supplementary File Table 11 shows that no contamination was observed in the BM-MSCs co-cultured with resting PBMCs and that <2% for those co-cultures with activated PBMCs.

Gene Expression data obtained from public repositories.

We used previous published and publicly available data from the Gene Expression Omnibus database. We focused in two studies showing gene expression of

(a) CD3 positive cells from PBMCs of healthy subjects (GSE31153)10 activated with anti-biotin magnetic beads pre-loaded with biotinylated antibodies against human CD2, CD3, and CD28, at a rate of 1 – 2 beads/cell, during 5 days and exposed to MSCs (in a 5 to 1 proportion).

(b) Adipose tissue MSCs (AT-MSCs; GSE18662)11 exposed to either pro-inflammatory cytokines (interferonγ, tumor necrosis factor α, and interleukine 6) or to a mixed lymphocyte reaction (MLR; separated with a Transwell culture plate).

Because in both studies, gene expression was analyzed using arrays, in order to compare the gene expression levels we categorized all expressed genes from each experiment in deciles. We considered low expressed genes those in the deciles 1 to 4, medium expressed those in deciles 5 to 7 and higher expressed those in deciles 8 to 10. Instead of analyzing all the expressed genes, we focused in those that were differentially expressed in our samples. Based on this categorization, 776, 179 and 303 DEGs were identified in BM-MSCs co-cultured with activated PBMCs.

First, we compared the level of expression of our BM-MSCs co-cultured with activated PBMCs with the expression levels of the AT-MSCs exposed to pro-inflammatory cytokines and to a MLR. We observed a higher correlation when compared with AT-MSCs exposed to pro-inflammatory cytokines that when exposed to MLR (n = 1,138) (rho = 0.61, p-value <10-4 versus rho = 0.56, p-value <10-4, respectively). A much lower correlation was observed with CD3+ T cells from PBMCs from healthy subjects, either activated (n = 1,044, rho = 0.15, p-value <10-4) or co-cultured with MSCs (n = 1,044, rho = 0.34, p-value <10-4). Supplementary File Tables 12 and 13 show a matrix formed with the deciles of expression of BM-MSCs cocultured with activated PBMCs and the deciles of expression of AT-MSCs cultured with pro-inflammatory cytokines or with a MLR, respectively. We observed that there were 21 genes (6 genes when compared with cytokines, 10 genes when compared with MLR and 5 when compared with both) that showed low expression levels in AT-MSCs and high expression levels in our samples (Supplementary File Table 14). Therefore, only 1.7% of the DEGs with high expression levels were, in turn, expressed at low levels in ATMSCs exposed to inflammation. Next, we assessed the expression levels of those 5 genes in CD3+ T cells from PBMCs of health subjects activated and exposed to MSCs. One gene showed low expression levels (MMP13: first deciles), two a medium expression level (ESM1: fifth deciles, LY75: seventh deciles), and two a high expression level (SERPINB4: eighth deciles, TRAF1: tenth deciles). Therefore only 0.7% of the DEGs highly expressed on BM-MSCs exposed to activated PBMCs showed a low expression level in AT-MSCs exposed to inflammatory stimuli and high expression levels in CD3+ T cells activated and exposed to MSCs.
